# The FAcilitates Chromatin Transcription complex regulates the ratio of glycolysis to oxidative phosphorylation in neural stem cells

**DOI:** 10.1093/jmcb/mjae017

**Published:** 2024-05-08

**Authors:** Yuhan Lou, Litao Wu, Wanlin Cai, Huan Deng, Rong Sang, Shanshan Xie, Xiao Xu, Xin Yuan, Cheng Wu, Man Xu, Wanzhong Ge, Yongmei Xi, Xiaohang Yang

**Affiliations:** Women's Hospital, Zhejiang University School of Medicine, Hangzhou 310058, China; Women's Hospital, Zhejiang University School of Medicine, Hangzhou 310058, China; CAS Key Laboratory of Brain Connectome and Manipulation, The Brain Cognition and Brain Disease Institute, Shenzhen Institute of Advanced Technology, Chinese Academy of Sciences, Shenzhen 518000, China; Institute of Genetics, Center for Genetic Medicine, The Fourth Affiliated Hospital, Zhejiang University School of Medicine, Yiwu 322000, China; Women's Hospital, Zhejiang University School of Medicine, Hangzhou 310058, China; Women's Hospital, Zhejiang University School of Medicine, Hangzhou 310058, China; Women's Hospital, Zhejiang University School of Medicine, Hangzhou 310058, China; Women's Hospital, Zhejiang University School of Medicine, Hangzhou 310058, China; Women's Hospital, Zhejiang University School of Medicine, Hangzhou 310058, China; Women's Hospital, Zhejiang University School of Medicine, Hangzhou 310058, China; Women's Hospital, Zhejiang University School of Medicine, Hangzhou 310058, China; Women's Hospital, Zhejiang University School of Medicine, Hangzhou 310058, China; Women's Hospital, Zhejiang University School of Medicine, Hangzhou 310058, China; Institute of Genetics, Center for Genetic Medicine, The Fourth Affiliated Hospital, Zhejiang University School of Medicine, Yiwu 322000, China; Women's Hospital, Zhejiang University School of Medicine, Hangzhou 310058, China; Institute of Genetics, Center for Genetic Medicine, The Fourth Affiliated Hospital, Zhejiang University School of Medicine, Yiwu 322000, China

**Keywords:** FAcilitates Chromatin Transcription complex, neural stem cell, *Ssrp, ERR*, glycolysis, oxidative phosphorylation

## Abstract

Defects in the FAcilitates Chromatin Transcription (FACT) complex, a histone chaperone composed of SSRP1 and SUPT16H, are implicated in intellectual disability. Here, we reveal that the FACT complex promotes glycolysis and sustains the correct cell fate of neural stem cells/neuroblasts in the *Drosophila* 3rd instar larval central brain. We show that the FACT complex binds to the promoter region of the estrogen-related receptor (*ERR*) gene and positively regulates *ERR* expression. ERR is known to act as an aerobic glycolytic switch by upregulating the enzymes required for glycolysis. Dysfunction of the FACT complex leads to the downregulation of *ERR* transcription, resulting in a decreased ratio of glycolysis to oxidative phosphorylation (G/O) in neuroblasts. Consequently, neuroblasts exhibit smaller cell sizes, lower proliferation potential, and altered cell fates. Overexpression of *ERR* or suppression of mitochondrial oxidative phosphorylation in neuroblasts increases the relative G/O ratio and rescues defective phenotypes caused by dysfunction of the FACT complex. Thus, the G/O ratio, mediated by the FACT complex, plays a crucial role in neuroblast cell fate maintenance. Our study may shed light on the mechanism by which mutations in the FACT complex lead to intellectual disability in humans.

## Introduction

Intellectual disability (ID), also known as mental retardation, is a neurodevelopmental disorder characterized by limitations in intellectual functioning and adaptive behaviors. The etiologies of ID are often linked to genetic and/or environmental factors that impact neurodevelopment (Vissers et al., 2016). In a clinical whole-exome sequencing study focusing on neurodevelopmental disorders, four patients with *de novo* missense variants of *SUPT16H* and one patient with a *de novo* deletion of *SUPT16H* were identified (Bina et al., 2020). The ClinVar database also documents a patient with neurodevelopmental disorders and epilepsy carrying a segment deletion containing *SSRP1*. Notably, SUPT16H and SSRP1 are two subunits of the heterodimeric FAcilitates Chromatin Transcription (FACT) complex in mammalian cells (Gurova et al., 2018). These observations suggest that defects in the FACT complex could be a genetic cause of ID. However, the mechanistic role of the FACT complex in neurodevelopment remains unknown.

While the FACT complex is implicated in the regulation of DNA replication, repair, and RNA transcription (Winkler et al., 2011), it is recognized as a histone chaperone. Within the FACT complex, SSRP1 binds to DNA through its DNA-binding motif, high mobility group (HMG)-box (Zhang et al., 2015), and SUPT16H interacts with histones and DNA (Winkler et al., 2011; Liu et al., 2020). More specifically, the FACT complex functions to dissociate one histone H2A–H2B dimer from the nucleosome, facilitating the passage of RNA polymerase II during transcription and subsequently reassembling the nucleosome (Orphanides et al., 1999; Belotserkovskaya et al., 2004; Winkler et al., 2011; McCauley et al., 2022). Therefore, the FACT complex is defined as a transcriptional elongation factor (Orphanides et al., 1999). It was reported that disruption of Ssrp1 could lead to embryonic lethality in a murine model (Cao et al., 2003). In zebrafish embryos, loss of Ssrp1a leads to defective DNA synthesis, impaired RNA transcription, and a prolonged S-phase of the cell cycle (Koltowska et al., 2013). The FACT complex is highly expressed in undifferentiated cells, while its expression level decreases as the cells undergo differentiation (Fazzio et al., 2008; Garcia et al., 2011). Thus, the FACT complex likely plays a crucial role in maintaining the undifferentiated state of cells.

*Drosophila* is a unique model for studying central nervous system (CNS) development. In *Drosophila, Ssrp* and *dre4* are ortholog genes of *SSRP1* and *SUPT16H*, respectively. *Ssrp* and *dre4* are highly expressed in the developing CNS (https://flybase.org/). Loss-of-function mutations in *Ssrp* or *dre4* caused developmental arrest at the larval stage (Sliter and Gilbert, 1992). It has been reported that clones of homozygous *Ssrp* mutant cells in compound eyes are far smaller than their wild-type counterparts, suggesting that *Ssrp* is required for normal cell proliferation (Quiquand et al., 2021). During the 3rd instar larval stage, neuroblasts (NBs) maintain an invariant size (10–11 μm in diameter) and volume after every asymmetric division, and the glucose metabolism in NBs primarily depends on glycolysis (Ito and Hotta, 1992; Homem et al., 2013, 2014). Starting from the early pupal stage, the main mode of glucose metabolism switches to oxidative phosphorylation, and NBs ‘shrink’ after asymmetric division (Homem et al., 2014). When the transcription factor Prospero enters the nucleus, these shrunken NBs undergo the final symmetrical division and produce two equal-sized daughter cells, terminating the NB cell fate (White and Kankel, 1978; Truman and Bate, 1988; Southall and Brand, 2009; Homem et al., 2014). Consequently, all NBs exit the cell cycle at the late stage of pupation, and no NBs are detectable in adult brains (Fernandez-Hernandez et al., 2013). Thus, changes in NB cell size are closely associated with cell fate alteration.

In this study, we reveal that the FACT complex is required for the maintenance of NB cell fate and correct brain development. *Ssrp* knockdown causes transcriptional downregulation of the estrogen-related receptor (*ERR*) gene (Eichner and Giguère, 2011; Tennessen et al., 2011; Cai et al., 2013; Kovalenko et al., 2019), leading to NB shrinkage and proliferation defect, smaller NB lineage size, and prolonged NB cell cycle. Overexpression of *ERR* or suppression of mitochondrial oxidative phosphorylation largely rescues these phenotypes. Changes in the metabolic rate of glycolysis or oxidative phosphorylation alter the ratio of glycolysis to oxidative phosphorylation (G/O) inside cells, which plays an important role in the maintenance of NB cell fate. Our data provide critical insight into the possible pathogenesis of human ID involving FACT complex mutations.

## Results

### Dysfunction of the FACT complex leads to neurodevelopmental defects associated with small-sized NBs

**Figure 1:**
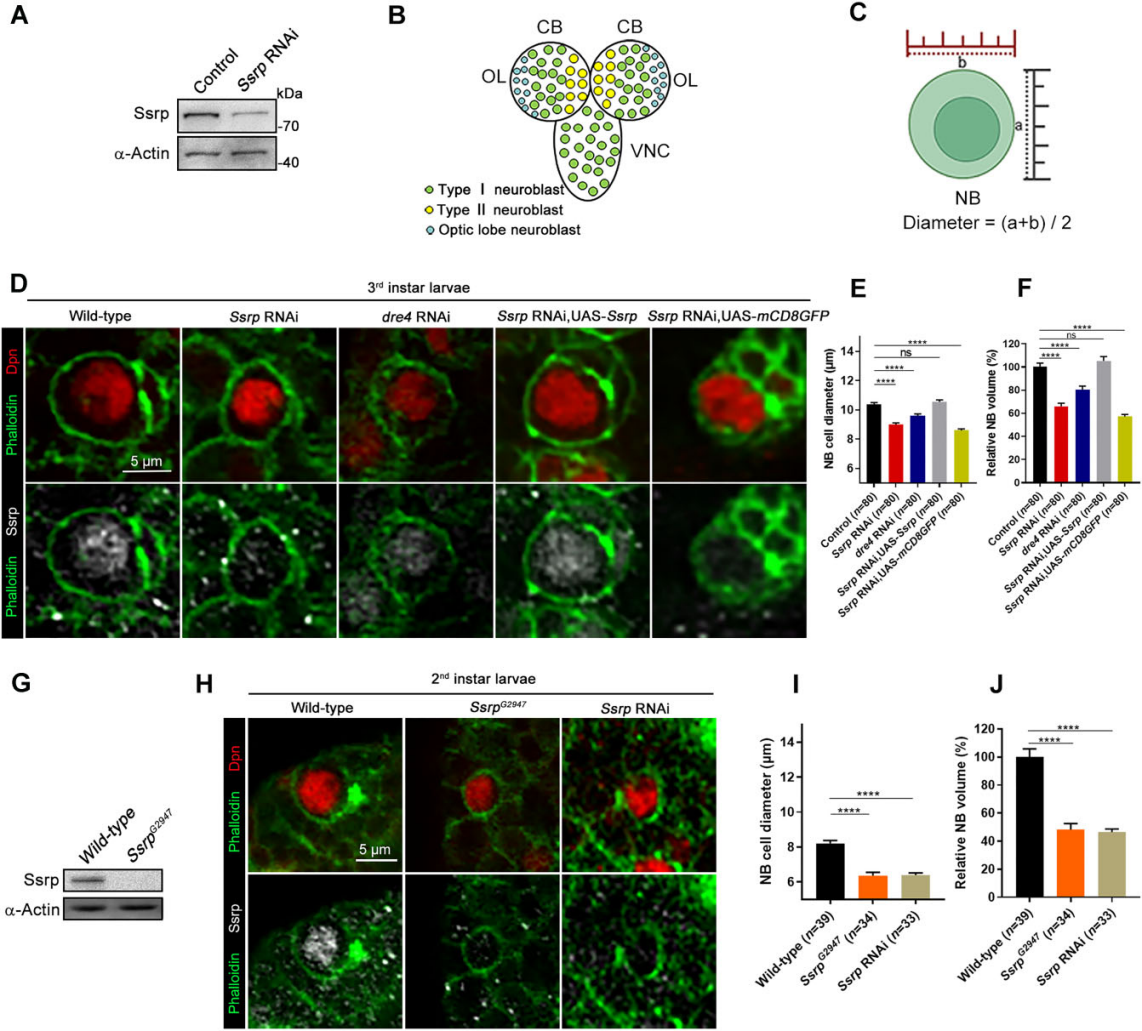
Dysfunction of the FACT complex leads to smaller NBs. (**A**) Western blot analysis of the Ssrp protein level in the 3rd instar larval brains of wild-type and *Ssrp*-knockdown flies. (**B**) Simplified diagram of *Drosophila* 3rd instar larval CNS showing different types of NBs and their locations. CB, central brain; OL, optic lobe; VNC, ventral nerve cord. (**C**) Measurement of NB diameters. (**D**) Confocal images of NBs in the 3rd instar larval brains labeled with anti-Dpn (red), anti-Ssrp (white), and phalloidin (green). Scale bar, 5 μm. (**E** and **F**) Statistical analyses of the NB diameters (**E**) and relative volumes (**F**) in **D**. (**G**) Western blot analysis of the Ssrp protein level in the 2nd instar larval brains of wild-type flies and *Ssrp^G2947^* mutants. (**H**) Confocal images of NBs in the 2nd instar larval brains labeled with anti-Dpn (red), anti-Ssrp (white), and phalloidin (green). Scale bar, 5 μm. (**I** and **J**) Statistical data of the NB diameters (**I**) and relative volumes (**J**) in **H**. The data were plotted as mean ± SEM. ****P < 0.0001; ns, no significant difference.

By searching the ClinVar database, we identified that the FACT complex genes *SSRP1* and *SUPT16H* are associated with human ID. To verify the potential link between the FACT complex and ID-related disease, we investigated *Drosophila* neurodevelopment in an RNAi line driven by *worniu*-GAL4 targeting the *Ssrp* coding region. The efficiency of *Ssrp* RNAi-mediated knockdown was confirmed by western blot analysis (Figure 1A). Adult *Drosophila* with *Ssrp* knockdown exhibited severe locomotion defects lying down on the bottom of the vials (Supplementary Video S1) and expired within 5 days after eclosion (median survival time = 3 days), a far shorter lifespan than that of wild-type *Drosophila* (median survival time = 57.5 days; Supplementary Figure S2).

There are two subtypes of NBs in the *Drosophila* central brain at the larval stage (Figure 1B). Since type I NBs represent the clear majority of the total neural stem cell population (Homem and Knoblich, 2012), we focused on this type of NBs in our study. The 3rd instar larval brains were triple-labeled with anti-Ssrp, anti-Deadpan (Dpn, a stem cell marker; San-Juan and Baonza, 2011), and phalloidin, which demarked the boundary of the cells. We examined NBs under a confocal microscope and found that the physical size of NBs after *Ssrp* knockdown tended to be smaller than that of their wild-type counterparts (Figure 1C and D). Quantitative analysis revealed that the average diameter of *Ssrp*-knockdown NBs was 8–9 μm, which was smaller than that of wild-type NBs (10–11 μm) (Figure 1E). Overall, the relative volume of NBs after *Ssrp* knockdown decreased by ∼34% (Figure 1F). Confocal images also revealed that the Ssrp protein was located in the nuclei of NBs (Figure 1D), which was consistent with previous reports (Shimojima et al., 2003; Swenson et al., 2016).

To eliminate the possibility of RNAi off-target effects, *Ssrp* or *mCD8GFP* was overexpressed in *Ssrp*-knockdown *Drosophila* (Figure 1D–F; Supplementary Figure S3). As expected, the reduced NB size by *Ssrp* knockdown was rescued by overexpression of *Ssrp* (Figure 1D–F). In addition, overexpression of *mCD8GFP* concurrent to *Ssrp* knockdown and *Ssrp* knockdown alone resulted in similar NB sizes (Figure 1D–F), indicating that the efficiency of GAL4 expression was not the rate-limiting factor, even with two UAS-elements in our study. We further employed the genetic mutant line *Ssrp^G2947^*, which contained a P-element inserted in the 5′UTR of *Ssrp* (Supplementary Figure S4). The Ssrp protein was undetectable in the NBs of homozygous *Ssrp^G2947^ Drosophila*, as determined by western blotting and immunofluorescence staining (Figure 1G and H), and the *Ssrp^G2947^* homozygotes died at the 2nd instar larval stage (48 h after egg laying). In *Ssrp^G2947^* mutant brains, the average diameter of the NBs was 6–7 μm, which was identical to that of *Ssrp*-knockdown NBs (6–7 μm) but smaller than that of wild-type NBs (8–9 μm) at the same developmental stage (2nd instar, 48 h after egg laying) (Figure 1I and J). Thus, the phenotype of small NB size was not due to any *Ssrp* RNAi-mediated off-target effects.

As the FACT complex is a heterodimer composed of Ssrp and Dre4 (Shimojima et al., 2003), we examined whether Ssrp acts together with Dre4 to fulfill its functions in brain development. We found that knockdown of *dre4* recapitulated the phenotypes mediated by *Ssrp* RNAi, such as small NB size, severe locomotion defects, and shorter lifespan (Figure 1D–F; Supplementary Video S1 and Figure S2), suggesting that Ssrp regulates the NB size as well as other phenotypes based on its role as a member of the FACT complex.

### Ssrp deficiency in the FACT complex inhibits NB proliferation and reduces NB lineage size

**Figure 2:**
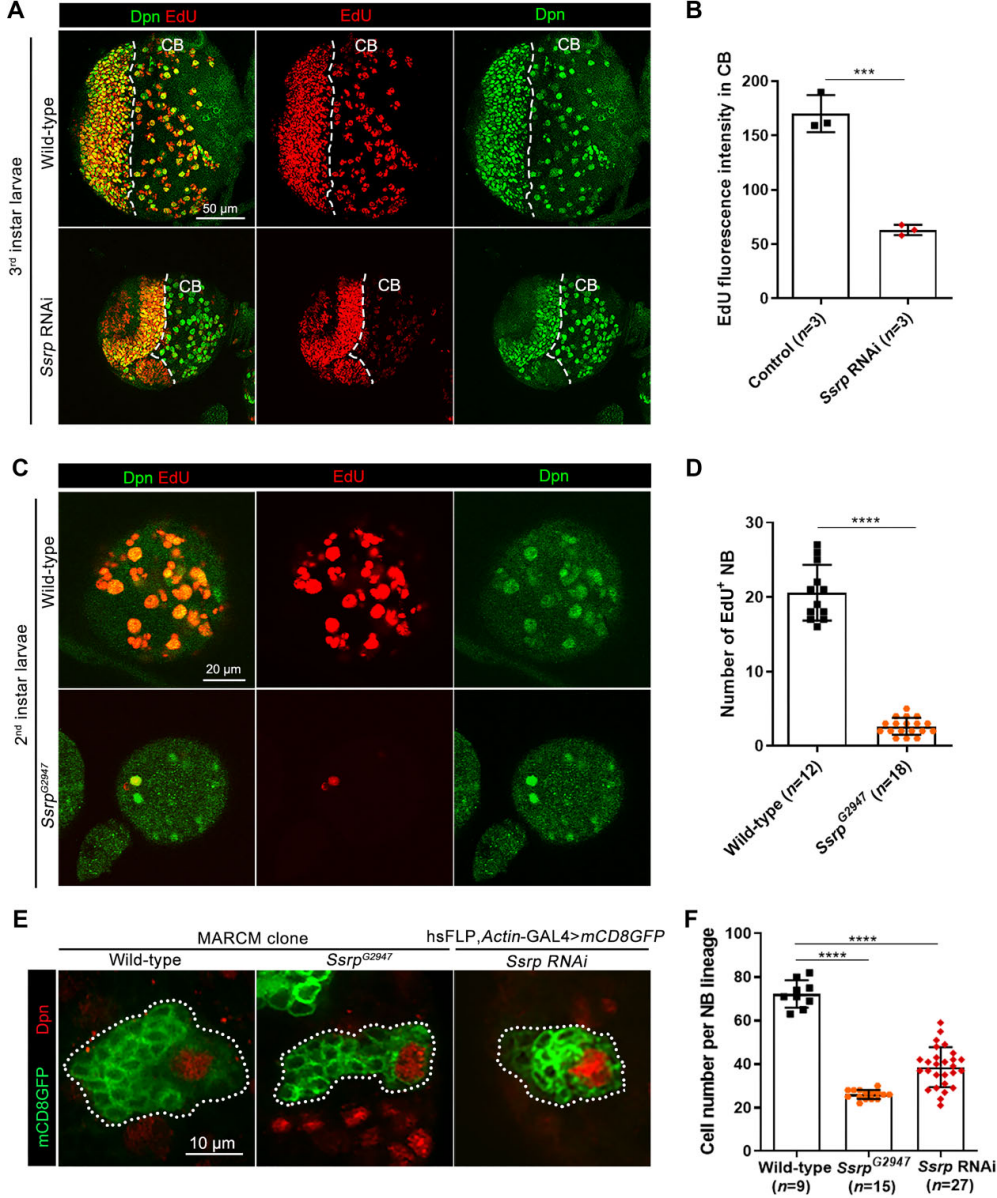
Deficiency of Ssrp causes NB proliferation defects and lineage shrinkage. (**A**) Confocal images of brain lobes in the 3rd instar larvae of wild-type and *Ssrp*-knockdown flies labeled with EdU (red) and anti-Dpn (green). CB, central brain. Scale bar, 50 μm. (**B**) Statistical analyses of EdU fluorescence intensities in **A**. (**C**) Confocal images of brain lobes in the 2nd instar larvae of wild-type flies and *Ssrp^G2947^* mutants labeled with EdU (red) and anti-Dpn (green). Scale bar, 20 μm. (**D**) Statistical analyses of the numbers of EdU^+^ NBs in **C**. (**E**) Confocal images of NB lineages in the 3rd instar larval brains of wild-type flies, *Ssrp*-knockdown flies, and *Ssrp^G2947^* mutants labeled with anti-Dpn (red) and mCD8GFP (green). Scale bar, 10 μm. (**F**) Statistical analyses of the average numbers of progeny cells per NB lineage in **E**. The data were plotted as mean ± SD. ****P < 0.0001; ***P < 0.001.

To investigate whether dysfunction of the FACT complex affects the proliferation of NBs, we carried out a standard EdU incorporation experiment. Fewer EdU signals were detected in NBs with *Ssrp* knockdown (Figure 2A and B), indicative of the disrupted cell cycle progression, while overexpression of *Ssrp* restored the EdU signals (Supplementary Figure S5A and B). Similarly, fewer EdU signals were detected in *Ssrp^G2947^* mutant brains (Figure 2C and D). In addition, the brain lobes of *Ssrp-*knockdown larvae were smaller than those of wild-type larvae (Figure 2A; Supplementary Figure S6A and B). These results suggested that *Ssrp* deficiency in the FACT complex disrupts the NB cell cycle, which in turn affects brain development. As expected, overexpression of *Ssrp* in *Ssrp*-knockdown *Drosophila* restored their brain lobe sizes (Supplementary Figure S6B), walking ability, and lifespans (median survival time = 59 days) similar to those of their wild-type counterparts (Supplementary Video S1 and Figure S2).

To determine NB proliferation potential, we performed the clonal expansion analysis, where a single NB and its progeny cells were labeled with mCD8GFP (outlining cell membranes). In *Ssrp*-knockdown brains and *Ssrp^G2947^* mutant brains, smaller clone sizes of the NB lineages were shown via mosaic analyses (Figure 2E). The average numbers of progeny cells per NB lineage of wild-type, *Ssrp*-knockdown, and *Ssrp^G2947^* flies were 72, 38, and 26, respectively (Figure 2F). Furthermore, in pupal brains (24 h after pupa formation, early pupal stage), the number of Dpn^+^ NBs was higher in *Ssrp-*knockdown *Drosophila* than in their wild-type counterparts (Supplementary Figure S7), suggesting a defect in NB cell fate termination.

### The FACT complex regulates the expression of the aerobic glycolytic switch ERR during brain development

**Figure 3:**
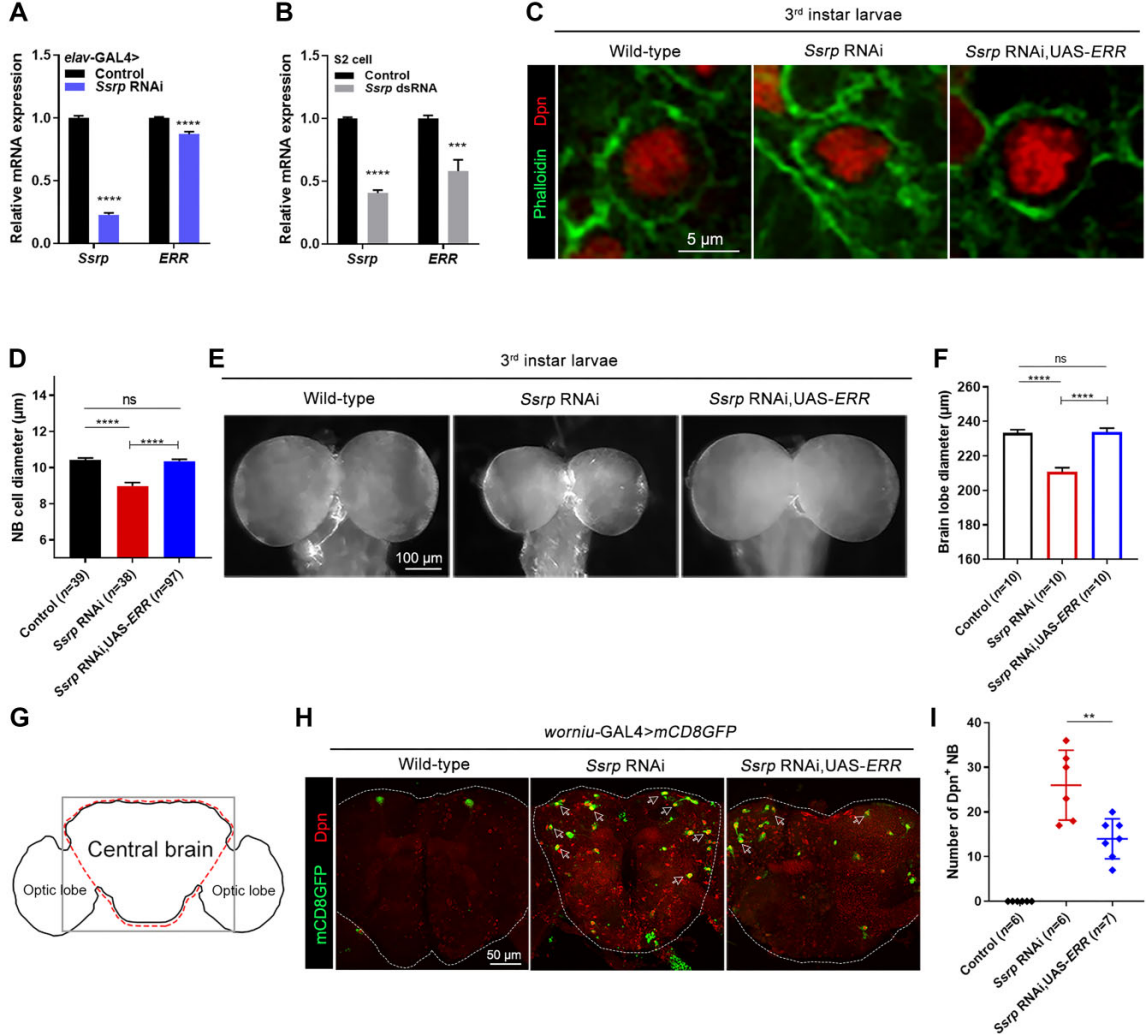
Overexpression of *ERR* rescues defective phenotypes in *Ssrp*-knockdown flies. (**A**) Relative mRNA expression levels of *Ssrp* and *ERR* in the 3rd instar larval brains of wild-type and *Ssrp*-knockdown flies driven by *elav*-GAL4, as determined by qPCR (*n* = 3). (**B**) Relative mRNA expression levels of *Ssrp* and *ERR* in wild-type and *Ssrp*-knockdown S2 cells (*n* = 3). (**C**) Confocal images of NBs in the 3rd instar larval brains labeled with anti-Dpn (red) and phalloidin (green). Scale bar, 5 μm. (**D**) Statistical analyses of the NB diameters in **C**. (**E**) Images of brain lobes in the 3rd instar larvae. Scale bar, 100 μm. (**F**) Statistical data of the brain lobe diameters in **E**. (**G**) Schematic diagram of the adult fly brain showing the positions of the central brain and optic lobes. (**H**) Confocal images of adult fly brains (1 day after eclosion) labeled with anti-Dpn (red) and mCD8GFP (green). Dpn^+^ NBs remained in adult brains are indicated by white arrows. Scale bar, 50 μm. (**I**) Statistical analyses of the number of Dpn^+^ NBs in **H**. The data were plotted as mean ± SEM (**A, B, D, F**) or mean ± SD (**I**). ****P < 0.0001; ***P < 0.001; **P < 0.01; ns, no significant difference.

It has been reported that NB sizes become smaller at the early pupal stage due to decreased levels of glycolysis and increased levels of oxidative phosphorylation (Homem et al., 2014). ERR acts as an aerobic glycolytic switch, which triggers and coordinates the transcriptional upregulation of nearly every single gene associated with glycolysis (Eichner and Giguère, 2011; Tennessen et al., 2011; Cai et al., 2013; Kovalenko et al., 2019). Thus, we wondered whether ERR level is affected by dysfunction of the FACT complex in the brain. Indeed, the transcriptional level of *ERR* was significantly downregulated in *Ssrp*-knockdown larval brains (Figure 3A). This result was further validated in *Drosophila* Schneider 2 (S2) cells upon *Ssrp* knockdown (Figure 3B), suggesting that ERR functions downstream of the FACT complex. Moreover, *ERR* overexpression in *Ssrp*-knockdown NBs in the larval brain completely rescued both NB size (Figure 3C and D) and brain lobe size (Figure 3E and F). Notably, *ERR* overexpression in wild-type NBs did not alter the cell size (Supplementary Figure S8). Overexpression of *ERR* in *Ssrp-*knockdown *Drosophila* significantly improved their lifespans (median survival time = 32.5 days; Supplementary Figure S2) and partially restored their walking ability (Supplementary Video S2).

An increased number of unterminated Dpn^+^ NBs were observed in *Ssrp*-knockdown pupal brains (Supplementary  Figure S7). To examine whether these abnormal NBs remained in adult brains, young adult fly brains (1 day after eclosion) were dissected and stained for Dpn^+^ NBs (Figure 3G). No Dpn^+^ NBs were detected in wild-type adult brains, whilst many Dpn^+^ NBs were found in the adult central brains of *Ssrp*-knockdown *Drosophila* (Figure 3H and I), suggesting that some NBs fail to be terminated upon dysfunction of the FACT complex and survive into adulthood. *ERR* overexpression in *Ssrp*-knockdown *Drosophila* significantly reduced the number of remaining Dpn^+^ NBs (Figure 3H and I), indicating that the termination of NB cell fate was partially restored. These findings support the proposal that the FACT complex controls *ERR* expression to regulate glycolysis in the brain.

### The FACT complex binds to the promoter region of ERR and positively regulates its expression

**Figure 4:**
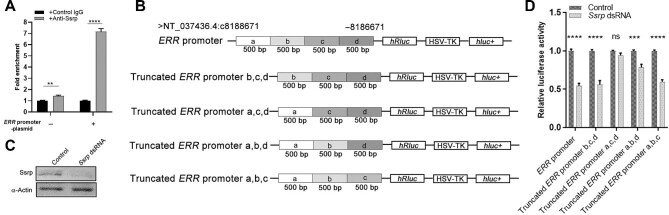
The FACT complex binds to the *ERR* promoter to promote *ERR* expression. (**A**) Enrichment of DNA fragments within the *ERR* promoter region with or without a plasmid containing the 2-kb promoter sequence, as determined by ChIP–qPCR (*n* = 3). (**B**) Constructions of dual-luciferase reporter plasmids (psi-CHECK-2) containing various 500-bp deletions within the *ERR* promoter (2 kb upstream of the 5′UTR of the *ERR* gene region). (**C**) Western blot analysis showing the decreased Ssrp protein level in *Ssrp*-knockdown S2 cells. (**D**) Statistical data of the relative luciferase activities in wild-type and *Ssrp*-knockdown S2 cells transfected with five dual-luciferase reporter constructs, respectively (*n* = 3). The data were plotted as mean ± SEM. ****P < 0.0001; ***P < 0.001; **P < 0.01; ns, no significant difference.

It is known that Ssrp contains an HMG-box DNA-binding motif (Winkler et al., 2011; Liu et al., 2020). To examine whether the FACT complex directly binds to the *ERR* promoter to regulate *ERR* expression, we performed chromatin immunoprecipitation (ChIP) in the *Drosophila* S2 cell line. We assumed that the 2-kb DNA fragment prior to the 5′UTR of the *ERR* gene region was the promoter and contained target sequences for the FACT complex. The anti-Ssrp antibody was able to pull down the genomic DNA sequence, which was recognized by quantitative polymerase chain reaction (qPCR) primers to lie ∼1.3 kb upstream of the 5′UTR of the *ERR* gene (Figure 4A), indicating the direct binding of Ssrp. Furthermore, in S2 cells transfected with plasmids containing this 2-kb promoter sequence, the anti-Ssrp antibody pulled down the promoter DNA fragment recognized with the same primers more effectively (7-fold enrichment compared to the control IgG, Figure 4A), confirming that the FACT complex binds directly to the promoter of *ERR* to regulate its expression.

Then, the dual-luciferase assay in S2 cells was employed, where Renilla luciferase was used as the experimental reporter and firefly luciferase was the control. To map the Ssrp target site(s) on the *ERR* promoter region, five dual-luciferase reporter constructs (psi-CHECK-2) were made, containing serial deletions within the *ERR* promoter (Figure 4B). S2 cells were transfected with dual-luciferase reporter constructs together with *Ssrp* dsRNA (Figure 4C). Luciferase activity was measured 48 h after transfection. We found that the relative luciferase activity representing *ERR* expression was reduced by half in S2 cells with the 2-kb *ERR* promoter in the presence of *Ssrp* dsRNA (Figure 4D), consistent with the previous observation that *Ssrp* knockdown reduced *ERR* expression. Similar results were observed when the first (a in Figure 4B) or the last (d in Figure 4B) 500-bp DNA fragment was deleted (Figure 4D). Sequential deletion of the second (b in Figure 4B) or the third (c in Figure 4B) 500-bp fragment within the *ERR* promoter only led to ∼5% or 20% reduction in luciferase activity, respectively (Figure 4D), suggesting that this 1-kb fragment, especially the second (b in Figure 4B) 500-bp fragment, is required for effective transcription of *ERR*. Based on ChIP and dual-luciferase assay data, we conclude that the FACT complex, or Ssrp, directly binds to the 1-kb fragment of *ERR* promoter, occurring 500 bp prior to the 5′UTR, to promote *ERR* transcription.

### The FACT complex controls the intracellular G/O ratio

It has been reported that the glucose metabolic pathway affects NB cell fate (Homem et al., 2014). Glycolysis is accompanied by NB self-renewal, whereas oxidative phosphorylation facilitates cell differentiation. It is possible that ERR acts as a controller to balance these two pathways in cells. Lower ERR levels in *Ssrp*-knockdown NBs could lead to lower glycolysis levels and, thus, a low G/O ratio.

**Figure 5:**
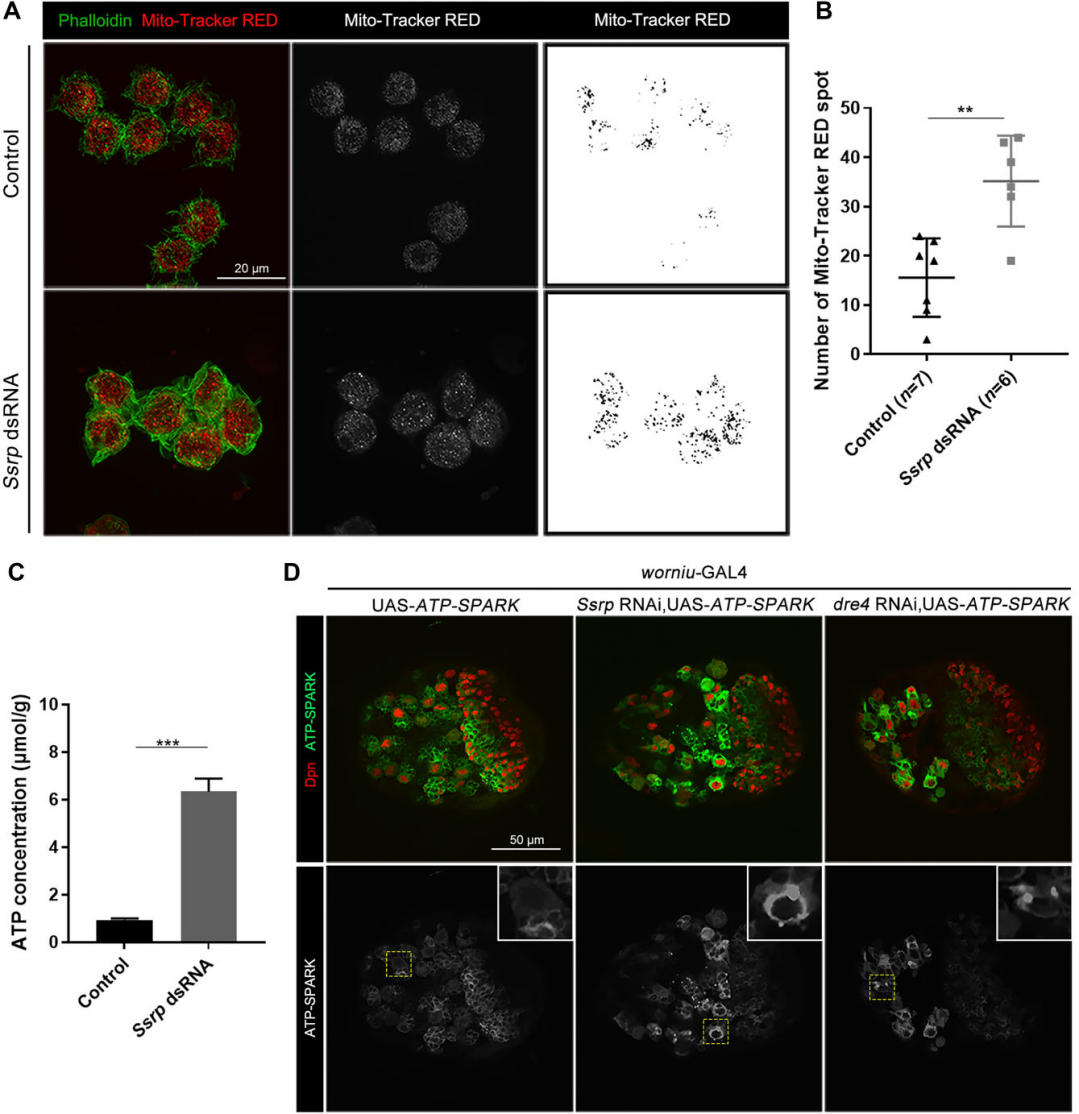
*Ssrp* knockdown leads to increased oxidative phosphorylation. (**A**) Confocal images of wild-type and *Ssrp*-knockdown S2 cells labeled with Mito-Tracker RED (red/white/black) and phalloidin (green). Scale bar, 20 μm. (**B**) Statistical analyses of the numbers of Mito-Tracker RED spots in **A**. (**C**) ATP concentration in S2 cells (*n* = 3). (**D**) Confocal images of the 3rd instar larval brains labeled with Dpn (red) and GFP-based ATP-SPARK (green/gray). A typical NB (within the yellow dashed frame) is enlarged to give a better view (within the white frame). Scale bar, 50 μm. The data were plotted as mean ± SD (**B**) or mean ± SEM (**C**). ***P < 0.001; **P < 0.01.

To explore whether dysfunction of the FACT complex alters oxidative phosphorylation status, we labeled mitochondria with Mito-Tracker RED in *Drosophila* S2 cells. Confocal microscopy demonstrated that the punctate mitochondrial signals in *Ssrp*-knockdown cells were significantly stronger than in the control wild-type cells (Figure 5A and B), indicating extra mitochondria emerging in the absence of Ssrp. Increased number of mitochondria usually suggests the higher level of oxidative phosphorylation and more ATP production in cells. Indeed, higher ATP levels were detected in *Ssrp*-knockdown S2 cells (Figure 5C). By using the ATP-SPARK method, which employs a GFP-based ATP reporter to show visible fluorescent droplets proportionally to the ATP level within cells (Zhang et al., 2018), we observed bright fluorescent droplets in larval NBs with *Ssrp* knockdown or *dre4* knockdown but not in wild-type larval NBs (Figure 5D), suggesting that ATP levels were increased in NBs upon dysfunction of the FACT complex. These results indicate that when glycolysis is suppressed in FACT complex-deficient flies, oxidative phosphorylation is upregulated, leading to a low G/O ratio.

### Suppression of oxidative phosphorylation rescues defective phenotypes in FACT complex-deficient flies

**Figure 6:**
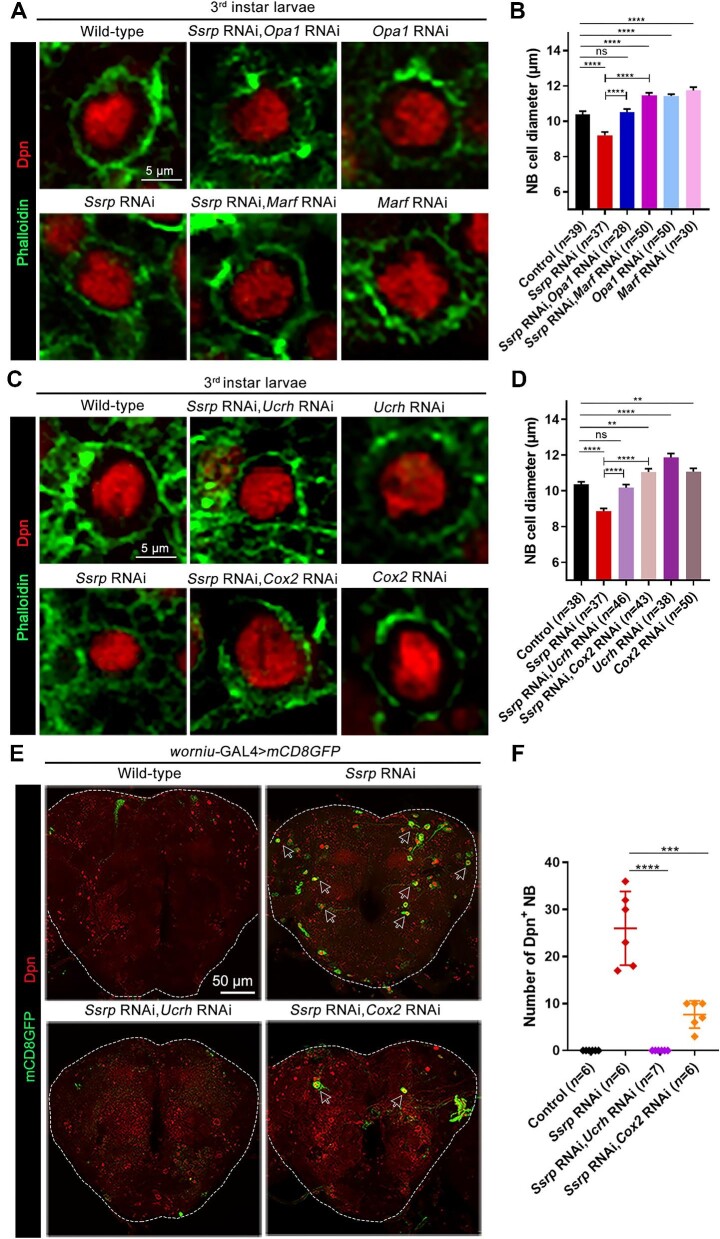
Suppression of either mitochondrial fusion or electron transfer in complex III/IV rescues defective phenotypes in *Ssrp*-knockdown flies. (**A** and **C**) Confocal images of NBs in the 3rd instar larval brains labeled with anti-Dpn (red) and phalloidin (green). Scale bar, 5 μm. (**B** and **D**) Statistical analyses of the NB diameters in **A** and **C**, respectively. (**E**) Confocal images of adult fly brains (1 day after eclosion) labeled with anti-Dpn (red) and mCD8GFP (green). Dpn^+^ NBs remained in adult brains are indicated by white arrows. Scale bar, 50 μm. (**F**) Statistical analyses of the numbers of Dpn^+^ NBs in **F**. The data were plotted as mean ± SEM (**B** and **D**) or mean ± SD (**F**). ****P < 0.0001, ***P < 0.001; **P < 0.01; ns, no significant difference.

Mitochondrial fusion is usually accompanied by a high level of oxidative phosphorylation (Mishra and Chan, 2016). We assumed that knocking down *Opa1* or *Marf*, the gene responsible for mitochondrial fusion (Sênos Demarco et al., 2019), would suppress oxidative phosphorylation. As expected, knockdown of *Opa1* or *Marf* in *Ssrp*-knockdown NBs completely restored NB sizes (Figure 6A and B), indicating that suppression of oxidative phosphorylation by preventing mitochondrial fusion restored NB sizes in FACT complex-deficient flies.

Similarly, knocking down the genes encoding subunits of protein complexes in the mitochondrial electron transfer chain, e.g. complex III (*Ucrh*) or complex IV (*Cox2*) also restored NB sizes in FACT complex-deficient flies (Figure 6C and D). The possible explanation was that the suppression of oxidative phosphorylation in *Ssrp*-knockdown NBs effectively raises the G/O ratio, which is critical for maintaining normal NB sizes. Notably, inhibition of oxidative phosphorylation in wild-type NBs (increased G/O ratio) led to even larger NB sizes, supporting the correlation between the G/O ratio and NB sizes (Figure 6A–D).

We next examined whether suppression of oxidative phosphorylation reduces the number of Dpn^+^ NBs in *Ssrp*-knockdown adult brains. Since knockdown of *Opa1* or *Marf* led to pupal lethality, we only studied the young adult flies with *Ucrh* knockdown or *Cox2* knockdown. In the adult brains, no remaining Dpn^+^ NBs were detected in *Drosophila* with *Ucrh* (complex III) knockdown, and the number of Dpn^+^ NBs decreased significantly upon *Cox2* (complex IV) knockdown (Figure 6E and F), suggesting that the termination of NB cell fate was largely restored.

In addition, suppression of either electron transfer or mitochondrial fusion did not rescue the smaller brain lobe sizes in *Ssrp-*knockdown larvae (Supplementary Figure S6), indicating that NB proliferation was not restored. Inhibition of electron transfer in *Ssrp*-knockdown *Drosophila* significantly improved their lifespans (median survival time = 45.5 days by *Ucrh* knockdown or 45 days by *Cox2* knockdown; Supplementary Figure S2). These flies also partially regained their walking ability (Supplementary Video S3).

Taken together, our data indicate that the FACT complex is involved in the maintenance of glycolysis levels in NBs. *Ssrp* knockdown leads to decreased *ERR* expression, resulting in a lower glycolysis level and a higher oxidative phosphorylation level, which brings down the cellular G/O ratio in NBs and causes defective NB phenotypes. Suppression of oxidative phosphorylation in *Ssrp*-knockdown NBs reverses the G/O ratio and restores NB sizes.

## Discussion

The FACT complex is a heterodimeric complex composed of SSRP1/Ssrp and SUPT16H/Dre4, with known involvement in the regulation of DNA replication, DNA repair, and RNA transcription (Winkler et al., 2011; Martin et al., 2018; Liu et al., 2020). Although several genetic mutations of SSRP1 and SUPT16H have been implicated in clinical cases associated with ID (Bina et al., 2020), the potential pathological mechanism has so far remained unclear. Our study using the *Drosophila* brain as a model reveals that the FACT complex controls the cellular G/O ratio in NBs, thereby affecting brain development.

At the *Drosophila* 3rd larval stage, the glucose metabolism in NBs is primarily through glycolysis (Homem et al., 2014). Under normal development, NBs maintain an invariant diameter and volume after every asymmetric division. However, at the early pupal stage, oxidative phosphorylation becomes dominant, and the renewed NBs begin to shrink after asymmetric divisions (Homem et al., 2014). Linking these two factors, the G/O ratio could be critical for the maintenance of NB size. Indeed, our data provide evidence that cell size and glucose metabolic status are coupled in NBs, and NB sizes are tightly controlled by the G/O ratio.

In our study, dysfunction of the FACT complex reduced transcriptional levels of *ERR*. The FACT complex directly interacted with the *ERR* promoter, facilitating *ERR* expression as a transcription elongation factor. A 1-kb DNA fragment, 500 bp prior to the 5′UTR of the *ERR* gene region, provided the potential binding site for the FACT complex, and the second 500-bp fragment (b in Figure 4B) exhibited more effective Ssrp regulatory effects in agreement with the search results in the JASPAR database that a DNA sequence of TCGTCGCTTTTCGT, the potential HMG binding motif, was identified within this 500-bp region.

It has been reported that SSRP1 is highly expressed in many cancers, including colorectal cancer (Wang et al., 2019; Wu et al., 2019), prostate cancer, lung cancer (Bi et al., 2019), bladder cancer (Song et al., 2021), breast cancer (Koman et al., 2012), malignant melanomas (Yu et al., 2021), hepatocellular carcinomas (Luo et al., 2021), and gliomas (Liao et al., 2017). High levels of SSRP1 in tumors are consistent with the consensus that glycolysis is dominant in cancer cells (Warburg effect) (Koppenol et al., 2011). Our study reveals that the FACT complex in NBs promotes glycolysis during neurodevelopment.

In S2 cells, dysfunction of the FACT complex increased mitochondrion number, accompanied by a higher ATP level, indicating elevated oxidative phosphorylation. This implies that when glycolysis is suppressed by *Ssrp* knockdown, oxidative phosphorylation can be upregulated by an innate mechanism inside cells. In other words, it is possible that multiple regulators are involved in the balancing of the G/O ratio in wild-type larval NBs.

Dysfunction of the FACT complex in the larval brain reduced NB proliferation potential. Overexpression of *ERR* restored both NB sizes and larval brain lobe sizes, whilst suppression of oxidative phosphorylation only restored NB sizes, but not brain lobe sizes, indicating that the NB proliferation potential was not restored. The possible explanation is that a sufficient level of glycolysis is required to generate the intermediate metabolites necessary for mitosis, while suppression of oxidative phosphorylation alone could not provide such metabolites, thus not restoring the ability of proliferation.

Dysfunction of the FACT complex altered the NB cell fate and disrupted the normal termination of NBs at the early pupal stage. Dpn^+^ NBs were detected in the adult brains of *Ssrp*-knockdown flies, indicating a prolonged NB cell cycle. Overexpression of *ERR* or suppression of oxidative phosphorylation in *Ssrp*-knockdown *Drosophila* reduced Dpn^+^ NBs in the adult brains. This implies that the G/O ratio also affects NB cell fate and lifespan.

**Figure 7:**
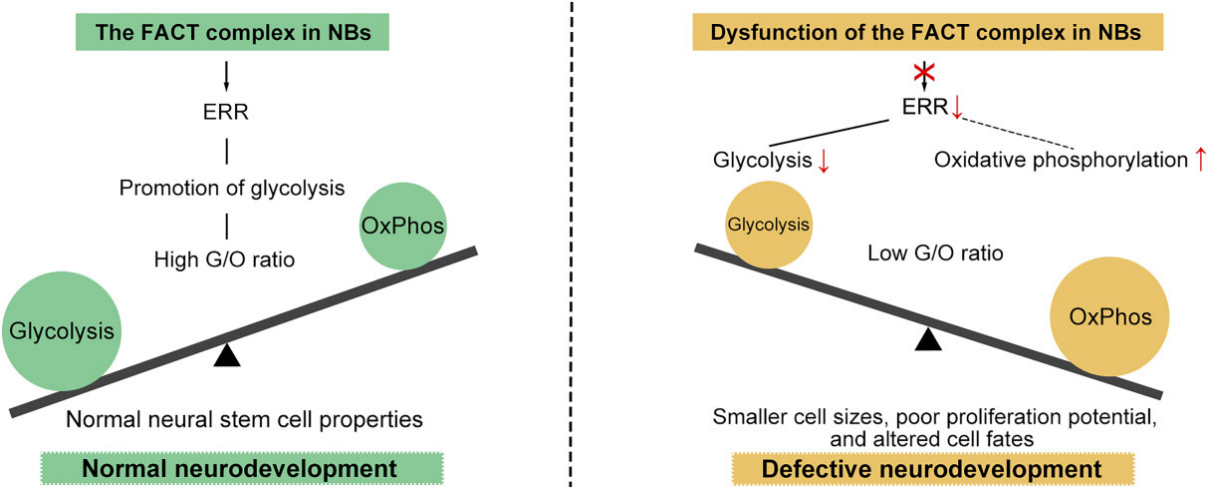
The role of the FACT complex in maintaining the cell fate of NBs during brain development. The FACT complex in NBs upregulates ERR and promotes glycolysis, resulting a high G/O ratio, to maintain the normal neural stem cell properties. Dysfunction of the FACT complex in NBs causes downregulation of ERR, resulting in a switch to oxidative phosphorylation and a low G/O ratio, which leads to smaller NB sizes, poor proliferation potential, and altered cell fates. During neurogenesis, any defects in NB cell properties likely lead to defective neurodevelopment and ID.

In conclusion, our study reveals a potential mechanism by which the FACT complex maintains the cell fate of NBs during brain development (Figure 7). In wild-type NBs, the FACT complex upregulates ERR and promotes glycolysis, resulting in a high G/O ratio, which ensures the normal neural stem cell properties of NBs and normal neurodevelopment. In NBs where the FACT complex is dysfunctional, ERR is downregulated, resulting in a metabolic switch from glycolysis to oxidative phosphorylation and thus a low G/O ratio. Consequently, the NBs exhibit smaller cell sizes, poor proliferation potential, and altered cell fates. During neurogenesis, any defects in NB cell properties would lead to defective neurodevelopment and ID. Our data shed light on the possible pathogenesis of ID disease caused by FACT complex mutations.

## Materials and methods

### Fly stocks and genetics

All flies were kept on standard corn medium at 25°C. The following fly stocks were used: *w^1118^* (THJ025, Tsinghua *Drosophila* Center), UAS-*Dicer2,worniu*-GAL4 (from our lab), UAS-*mCD8GFP,worniu*-GAL4 (from our lab), *elav*-GAL4 (from our lab), *If*/*CyO-GFP* (from our lab), *Ssrp* RNAi (THU2221, Tsinghua *Drosophila* Center), *Ssrp^G2947^/CyO* (29686, Bloomington *Drosophila* Stock Center), *dre4* RNAi (THU1558, Tsinghua *Drosophila* Center), UAS-*Ssrp* (F001903, Zurich ORFeome Project), UAS-*ERR* (83690, Bloomington *Drosophila* Stock Center), *ERR* RNAi (THU2474, Tsinghua *Drosophila* Center), *Opa1* RNAi (THU0811, Tsinghua *Drosophila* Center), *Marf* RNAi (TH02740.N, Tsinghua *Drosophila* Center), *Ucrh* RNAi (TH04741.N, Tsinghua *Drosophila* Center), and *Cox2* RNAi (TH04769.N, Tsinghua *Drosophila* Center).

Mosaic analysis with a repressible cell marker (MARCM) was performed as previously described (Lee and Luo, 2001; Sang et al., 2022). For *Ssrp^G2947^* clones, the fly line with the genotype of *FRT42D tub*-GAL80/*CyO*;*hsFLP,elav*-GAL4,UAS-*mCD8GFP* was used as the tool. *FRT42D Ssrp^G2947^*/*CyO-GFP* was obtained by homologous recombination of lines *FRT42D* (1802, Bloomington *Drosophila* Stock Center) and *Ssrp^G2947^*/*CyO-GFP*. The MARCM clones were GFP-labeled. For FLP-out mosaic analysis, the fly line with the genotype of *hsFLP*;*act*>*y*^+^>GAL4,UAS-*mCD8GFP* was crossed with RNAi lines (Germani et al., 2018). Both MARCM clone and FLP-out clone analyses were carried out upon a 45-min heat-shock treatment at 37°C for *Drosophila* at 36 h after egg laying.

### Antibody generation

A fusion protein containing 283 amino-acid residues from the C-terminus of Ssrp was used to generate a polyclonal antibody in rabbits (Supplementary Figure S1). The fusion protein sequence was cloned into a pGEX-4T-1 vector and expressed in *Escherichia coli*. After purification, the fusion protein was used to generate antisera with the standard protocol.

### Immunofluorescence staining

Larval and adult brains at different stages were dissected in Schneider's *Drosophila* Medium (Gibco). Samples were fixed for 15 min in phosphate-buffered saline (10 mM NaH_2_PO_4_/Na_2_HPO_4_ and 175 mM NaCl, pH 7.4) with 4% paraformaldehyde at room temperature (Wu et al., 2018; Sang et al., 2022). The samples were blocked with 1% bovine serum albumin and incubated with primary antibodies at 4°C overnight and then washed three times. The commercial secondary antibodies were added to the samples for 1-h incubation, or phalloidin was added for 20-min incubation at room temperature. Anti-fade Mounting Medium (P0126, Beyotime) was used to prevent the bleaching of the fluorescent signals. For the EdU incorporation experiment, Click-iT EdU Imaging Kits (Alexa Fluor 555) (C10338, Invitrogen) were used for live samples incubated with EdU for 1 h before fixing. For Mito-Tracker staining, S2 cells were mixed with Mito-Tracker RED (9082, Cell Signaling Technology) for 40 min. The images were obtained using an Olympus FV1000 confocal microscope and processed using ImageJ and Adobe Photoshop.

The following primary antibodies were used: rabbit anti-Ssrp (1:2000, generated in this study), guinea pig anti-Dpn (1:1000, a gift from Yu Cai, Temasek Life Sciences Laboratory), and chicken anti-GFP (1:1000, ab13970, Abcam). All commercial secondary antibodies were from the Jackson Laboratory. F-actin was stained with Alexa Fluor™ 488 phalloidin (A12379, Invitrogen) at 1:2000.

### Western blotting

The 3rd instar larval brains were collected and homogenized in RIPA lysis buffer [50 mM Tris-HCl, pH 8.0, 150 mM NaCl, 1 mM EDTA, 1% Triton X-100, and 0.5% sodium dodecyl sulfate (SDS)] containing cOmplete^TM^ protease inhibitor cocktail (4693132001, Roche) at 4°C for 30 min. S2 cells were lysed in RIPA lysis buffer at room temperature for 5 min. The samples were subjected to SDS–polyacrylamide gel electrophoresis and transferred to a polyvinylidene fluoride membrane. Rabbit anti-α-Actin (1:1000, 23660-1-AP, Proteintech) and rabbit anti-Ssrp (1:2000) were used as primary antibodies, with horseradish peroxidase-conjugated anti-rabbit (1:5000, Abcam) as the secondary antibody.

### qPCR

RNA was extracted using the TRIzol Kit (Invitrogen). A HiScript II 1st Strand cDNA Synthesis Kit (R211-01, Vazyme) was used for reverse transcription according to the manufacturer's protocol. qPCR was carried out in the Bio-Rad CFX-96 PCR System with ChamQ SYBR qPCR Master Mix (Q311-02, Vazyme) in 96-well plates. The following primers were used: *rp49* forward: 5′-GCTAAGCTGTCGCACAAA-3′; *rp49* reverse: 5′-TCCGGTGGGCAGCATGTG-3′; *ERR* forward: 5′-ACTAATGGGCATGCTCAAGGA-3′; and *ERR* reverse: 5′-TATCTTGACATCGCACAGCG-3′.

### S2 cell culture and transfection

*Drosophila* S2 cells were cultured in Schneider's medium (21720-001, Gibco) supplemented with 10% fetal bovine serum (10099-141, Gibco) at 25°C. *Ssrp* dsRNA was transcribed from a cDNA fragment containing the C-terminal 400 bp of *Ssrp* with MEGAscript® RNAi Kit (AM1626, Invitrogen). S2 cells were transfected with the *Ssrp* dsRNA using Effectene Transfection Reagent (301425, QIAGEN).

### ATP assay

S2 cells (cultured in 6-well plates) were collected into centrifuge tubes. An ATP Assay Kit (S0026, Beyotime) was used for ATP concentration measurement according to the manufacturer's protocol.

### ChIP analysis

ChIP analysis was performed using Sonication ChIP Kits (RK20258, ABclonal). S2 cells were fixed with 1% formaldehyde at 25°C for 10 min. All ChIP experimental steps followed the protocol recommended by the manufacturer. DNA was purified by the MinElute PCR Purification Kit (QIAGEN). The pulled-down material and input DNA were prepared for qPCR analysis with the following primers around the *ERR* promoter region: forward: 5′-GTGGCACACCTTGAGCTTGC-3′; reverse: 5′-GAGGCTACAGCCTGTACAGCCT-3′.

### Dual-luciferase assay

The dual-luciferase assay was carried out in S2 cells. psi-CHECK-2, a dual-luciferase reporter construct containing firefly and Renilla luciferase, was employed in this assay. A 2-kb DNA fragment prior to the 5′UTR of the *ERR* gene region was cloned into psi-CHECK-2 as an *ERR* promoter. Four additional fragments with 500-bp sequential deletions within the 2-kb *ERR* promoter were cloned into psi-CHECK-2, respectively. S2 cells were transfected with individual constructs, with or without *Ssrp* dsRNA. After 48 h, S2 cells were lysed for luciferase activity using a Dual-Luciferase® Reporter Assay System (E1910, Promega).

### Survival rate analysis

Adult flies were kept in vials with the standard medium at 25°C. Three vials, each containing 10 adult flies, were used. The experiment was repeated three times. Surviving flies were counted every 24 h. The data were processed using Gehan–Breslow–Wilcoxon test in GraphPad Prism.

### Locomotion assay

Groups of 10 female flies were placed in empty vials, and the vials were tapped to bring the flies to the bottom of the vial. The motor abilities of the flies were then evaluated based on videos.

### Statistical analysis

The data of survival rate analysis were processed using the Gehan–Breslow–Wilcoxon test in GraphPad Prism. The other statistical data were processed using unpaired, two-tailed Student's *t*-test in GraphPad Prism.

## Supplementary Material

mjae017_Supplemental_Files

## Notes

**Author contributions:** Y.L., L.W., X.Y., and Y.X. conceived the project and designed the experiments. Y.L., L.W., W.C., H.D., and R.S. performed the experiments. Y.L. and S.X. analysed the data. Y.L., X.X., Xin Yuan, C.W., M.X., and W.G. contributed to the project discussion and coordination. Y.L. wrote the initial manuscript. Y.L., L.W., X.Y., Y.X., C.W., and H.D. revised the manuscript. All the authors read and approved the final manuscript.
