# Supplementary material for: The FAcilitates Chromatin Transcription complex regulates the ratio of glycolysis to oxidative phosphorylation in neural stem cells
Source: J Mol Cell Biol. 2024 May 8;16(4):mjae017. doi: 10.1093/jmcb/mjae017 (PMC11467811; doi:10.1093/jmcb/mjae017)
Supplement: mjae017_Supplemental_Files [file mjae017_supplemental_files.zip › JMCB-2023-0138.R3_Supplementary material.pdf]

Supplementary material

The FAcilitates Chromatin Transcription complex regulates the ratio of glycolysis to oxidative phosphorylation in neural stem cells

Yuhan Lou[1,†], Litao Wu[1,3,†], Wanlin Cai[2], Huan Deng[1], Rong Sang[1], Shanshan Xie[1], Xiao Xu[1], Xin Yuan[1], Cheng Wu[1], Man Xu[1], Wanzhong Ge[1], Yongmei Xi[1,2,*], and Xiaohang Yang[1,2,*]

Supplementary Figures

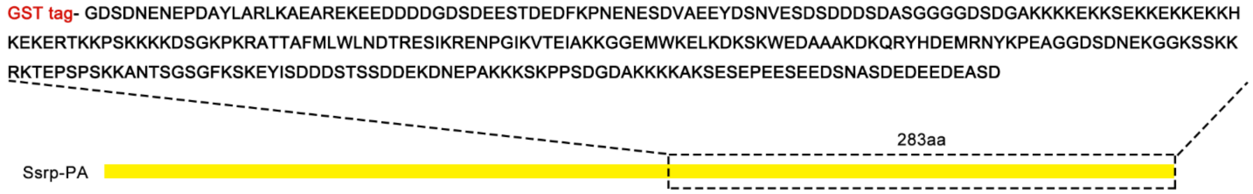

Supplementary Figure S1 The diagram showing the fusion protein sequence containing 283 amino acid residues from C-terminal of Ssrp for antibody generation.

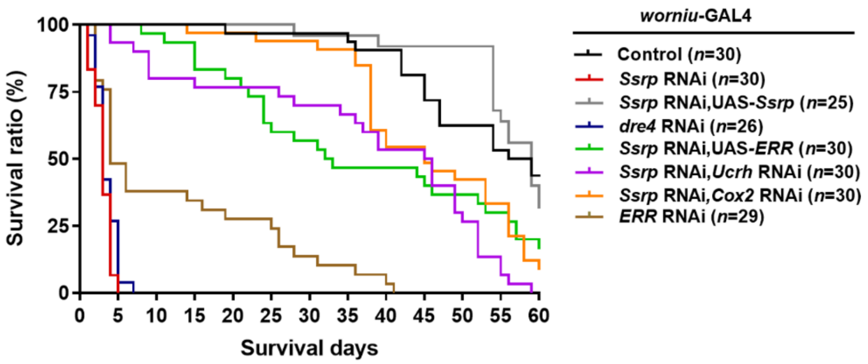

P value (Gehan-Breslow-Wilcoxon test)	control	*Ssrp* RNAi
control	/	<0.0001
Ssrp RNAi	<0.0001	/
Ssrp RNAi,UAS-*Ssrp*	0.5292(ns)	<0.0001
dre4 RNAi	<0.0001	0.2275(ns)
Ssrp RNAi,UAS-*ERR*	0.0021	<0.0001
Ssrp RNAi, *Ucrh* RNAi	<0.0001	<0.0001
Ssrp RNAi, *Cox2* RNAi	0.0039	<0.0001
ERR RNAi	<0.0001	0.0003

Supplementary Figure S2 Survival curves of adult flies with various treatments. Triplicated samples with 10 adult female flies in each vial were kept in 25℃ and flies were transferred to new vials every other day, *n*=30. The data were processed using Gehan-Breslow-Wilcoxon test (survival analyses) in GraphPad Prism.

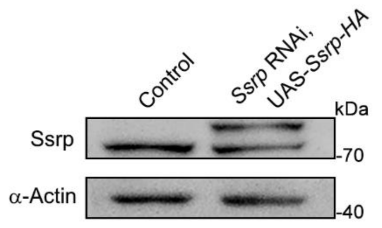

Supplementary Figure S3 Conformation of HA-tagged Ssrp protein expression in 3rd instar larval brains of *Ssrp* rescued animals.

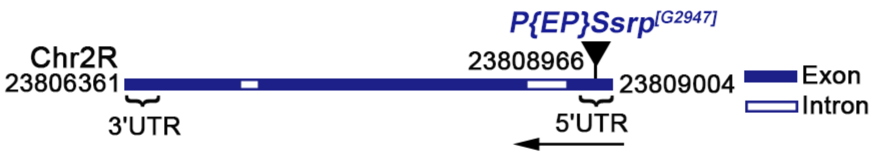

Supplementary Figure S4 Genomic organization of *Ssrp*^{G2947} gene. The mutation carried an EP element inserted into 5'UTR region of *Ssrp* (2R:23808966).

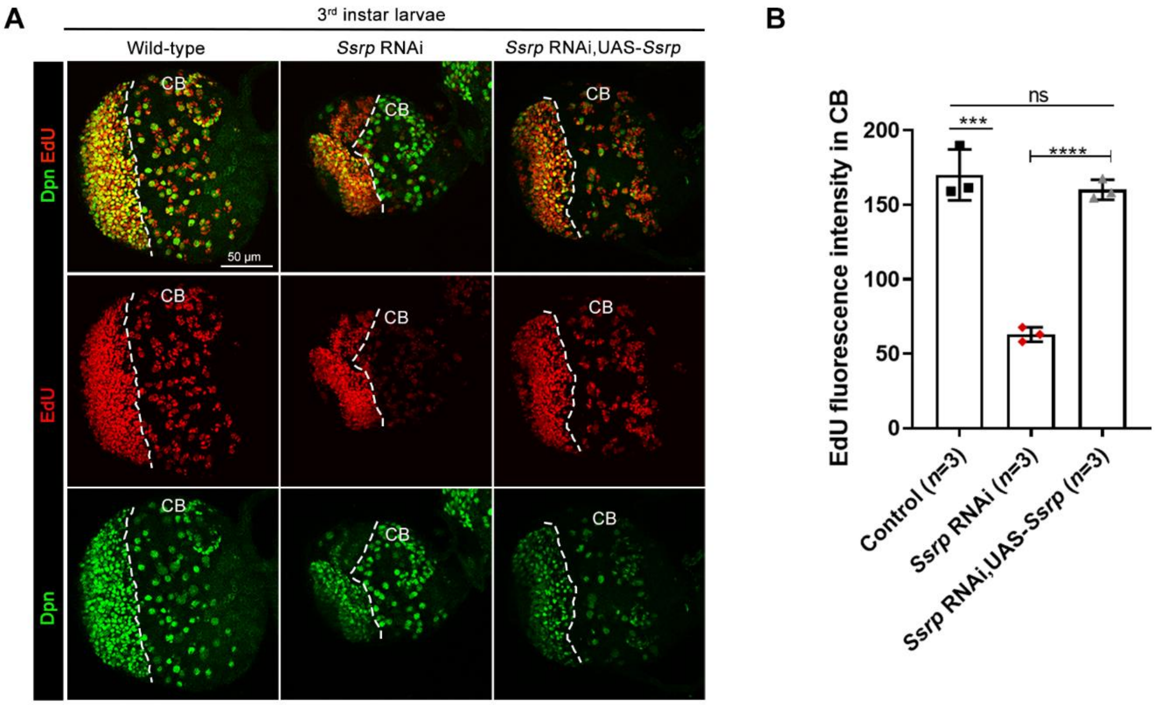

Supplementary Figure S5 Overexpression of *Ssrp* rescued EdU incorporation. (**A**) Confocal brain lobe images of the 3rd instar larvae of the wild type, *Ssrp* RNAi and *Ssrp* RNAi with UAS-*Ssrp* double labeled with EdU (red) and anti-Dpn (green). CB: central brain. *worniu*-Gal4 was used in the experiments. Scale bars: 50 μm. (**B**) Statistical data of EdU fluorescence intensity in the 3rd instar larval central brains by Image J. The data were plotted as mean ± SD. ***$P < 0.001$, ****$P < 0.0001$, ns=no significant difference.

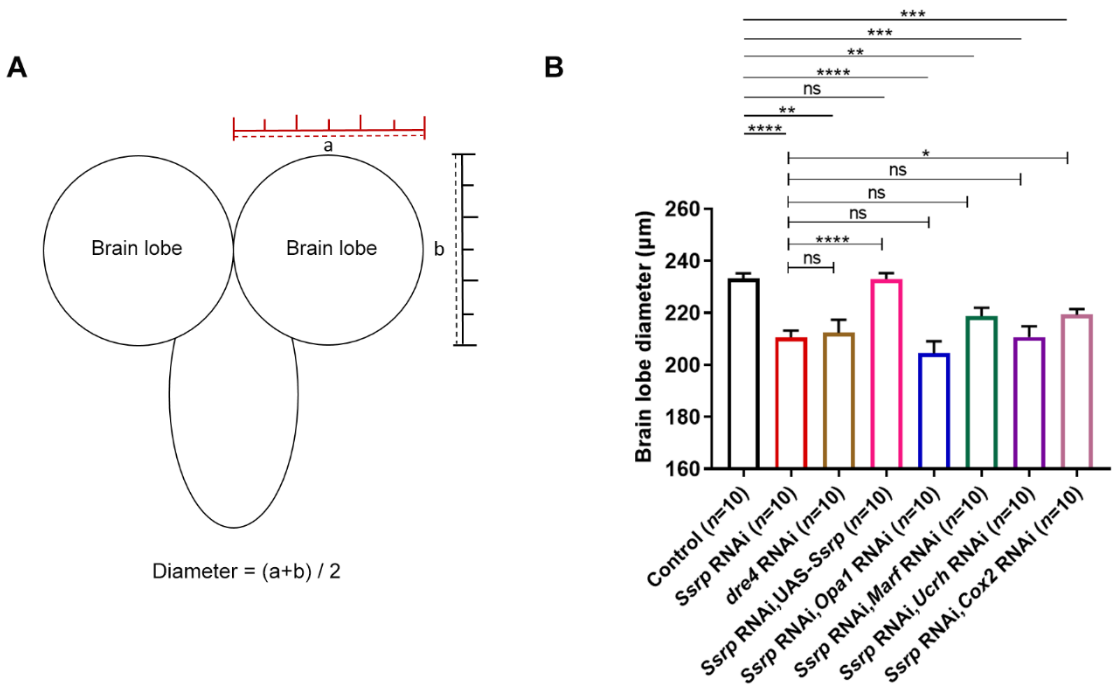

Supplementary Figure S6 Brain lobe diameters in the 3rd instar larvae. (**A**) Measurement of brain lobe diameters. (**B**) Statistical data of the brain lobe diameters. The data were plotted as mean ± SEM. *P < 0.05, **P < 0.01, ***P < 0.001, ****P < 0.0001, ns=no significant difference.

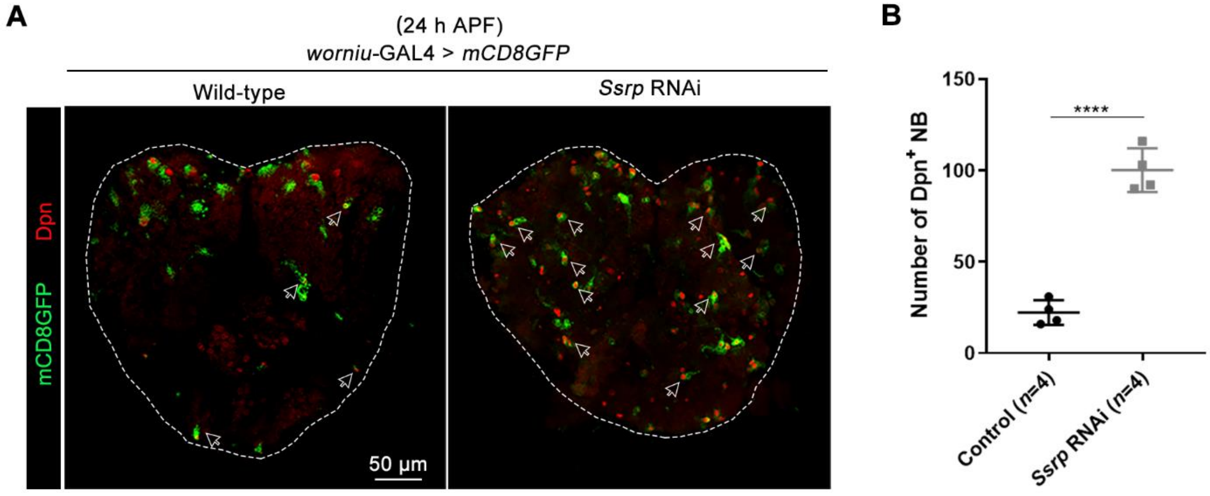

Supplementary Figure S7 The numbers of Dpn+ NBs were higher in *Ssrp* knockdown animals than in the wild type counterparts in the pupal brain. (**A**) Confocal images of pupal brains (24 h after pupa formation/APF) of the wild type and *Ssrp* RNAi treatment. Scale bars: 50 μm. **B** Statistical data of the quantity of Dpn+ NBs in pupal brains. The data were plotted as mean ± SD. ****P < 0.0001.

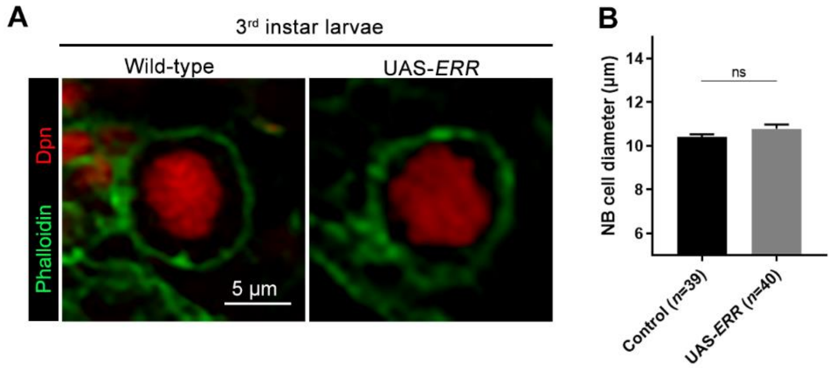

Supplementary Figure S8 *ERR* overexpression in the wild type NBs did not alter the cell sizes. (**A**) Confocal images of NBs in the 3rd instar larval brains of the wild type and *ERR* overexpression, double labeled with anti-Dpn (red) and Phalloidin (green). *worniu*-Gal4 was used in the experiments. Scale bars: 5 μm. (**B**) Statistical data of the NB cell diameters in the 3rd instar larval brains. The data were plotted as mean ± SEM. ns=no significant difference.

Supplementary Videos

Supplementary Video S1 Locomotion assay of adult flies of the wild type, *Ssrp* RNAi, *dre4* RNAi and *Ssrp* RNAi with UAS-*Ssrp*. Adult flies with *Ssrp* knockdown or *dre4* knockdown exhibited severe locomotion defects. Adult flies with *Ssrp* overexpression in *Ssrp* knockdown background regained walking abilities.

Supplementary Video S2 Locomotion assay of adult flies of the wild type, *Ssrp* RNAi and *Ssrp* RNAi with UAS-*ERR*. *Ssrp* knockdown flies with *ERR* overexpression partially regained walking abilities.

Supplementary Video S3 Locomotion assay of adult flies of the wild type, *Ssrp* RNAi, *Ssrp* and *Ucrh* (compex Ⅲ) double RNAi, and *Ssrp* and *Cox2* (compex Ⅳ) double RNAi. *Ssrp* and *Ucrh* double knockdown or *Ssrp* and *Cox2* double knockdown flies partially regained walking abilities.
